# Antibacterial Mechanism of Dipicolinic Acid Against *Xanthomonas citri* pv. *glycines* and Its Efficacy for the Management of Soybean Bacterial Pustule Disease

**DOI:** 10.3390/biom16040605

**Published:** 2026-04-19

**Authors:** Lei Chen, Jia-Xuan Shen, Ming-Yi Zhang, Xin-Chi Shi, Lei Xu, Si-Yuan Liu, Daniela D. Herrera-Balandrano, Pere Clapés, Jie Gong, Dong Liu, Su-Yan Wang, Pedro Laborda

**Affiliations:** 1 School of Life Sciences, Nantong University, Nantong 226019, China; lei_chen@stmail.ntu.edu.cn (L.C.); 2309110247@stmail.ntu.edu.cn (J.-X.S.); 2309110229@stmail.ntu.edu.cn (M.-Y.Z.); shxch0301@ntu.edu.cn (X.-C.S.); jgong188@ntu.edu.cn (J.G.); tom@ntu.edu.cn (D.L.); 2 Core Facilities, Faculty of Health Science, Nantong University, Nantong 226019, China; xl2005@ntu.edu.cn; 3 Nantong University Analysis & Testing Center, Nantong University, Nantong 226019, China; lsiyuan@ntu.edu.cn; 4 Department of Food and Human Nutritional Sciences, University of Manitoba, Winnipeg, MB R3T 2N2, Canada; daniela.herrerabalandrano@umanitoba.ca; 5 Department of Biological Chemistry, Institute for Advanced Chemistry of Catalonia (IQAC-CSIC), 08034 Barcelona, Spain; pere.clapes@iqac.csic.es

**Keywords:** antibacterial metabolites, foliar diseases, bacterial membrane, biofilm inhibitors, biological control

## Abstract

*Bacillus* species are extensively studied, utilized, and commercialized biocontrol agents, demonstrating significant effectiveness in managing a variety of plant diseases. *Bacillus* possesses a robust intrinsic biosynthetic ability, capable of producing a diverse array of antimicrobial metabolites, including dipicolinic acid (DPA; 2,6-pyridinedicarboxylic acid), which exhibits antifungal properties and serves as a principal structural component of *Bacillus* spores. This study revealed that DPA exhibits significant antibacterial activity against the hazardous soybean pathogen *Xanthomonas citri* pv. *glycines* (*Xcg*), with an EC_50_ value of 53.2 μg/mL. DPA inhibited *Xcg* swimming motility, extracellular protease activity, and biofilm formation, while inducing significant membrane irregularities in *Xcg* cells. DPA treatment downregulated the expression of several *Xcg* membrane integrity-related genes, including *cirA*, *czcA*, *czcB*, *emrE*, and *tolC*. The preventive and curative application of 500 μg/mL DPA reduced *Xcg* symptoms by 82.7% and 83.8%, respectively, and induced the accumulation of the isoflavone genistin in soybean leaves. DPA exhibited only weak toxicity in the zebrafish model, suggesting its potential suitability for agricultural commercialization. Overall, this study provides the first detailed characterization of the antibacterial mechanism of DPA against a phytopathogenic bacterium, *Xcg*, and identifies DPA as a previously underexplored antibacterial metabolite from *Bacillus* and *Paecilomyces* with potential for disease management.

## 1. Introduction

*Xanthomonas* is a large genus of Gram-negative bacteria that causes foliar and vascular diseases on different crops [1]. *Xanthomonas citri* pv. *glycines* (*Xcg*; formerly known as *X. axonopodis* pv. *glycines* and *X. campestris* pv. *glycines*) causes bacterial pustule disease in soybean [2]. *Xcg* infection results in brown pustules on soybean leaves surrounded by yellow haloes, leading to premature defoliation. Several outbreaks of bacterial pustule disease have occurred in China, South Korea, and Thailand, generating significant losses in soybean production [3,4]. Bacterial pustule disease in South Korea resulted in yield losses of 19.8% in 2006 and 16.8% in 2007 [5]. Over the past few decades, yield losses due to soybean bacterial pustule disease in Thailand were reported to range from 20% to 35% of the total soybean production [6].

Several key pathogenicity factors have been identified in *Xcg* [7], including type III secretion systems (T3SS) [3,5], the phosphate uptake systems phoB and phoU [8], the LysR-type transcriptional regulator LcrX [9], and the outer membrane protein OmpA [10]. *Xcg* can form a dense biofilm, which is rich in proteases and is involved in *Xcg* pathogenicity [11,12]. *flgK* and *pilD* genes, which are involved in flagellum and pili formation, respectively, have also been reported to play a key role in *Xcg* virulence [13].

While *Xanthomonas* has been traditionally controlled using copper- and zinc-based bactericides [14,15], the detrimental effects of heavy metals on human health and the environment have led to restrictions on their agricultural use [16,17]. This situation has prompted the development of alternative strategies for the management of bacterial pustule disease. Despite the use of breeding to develop resistant soybean varieties, none of the reported varieties showed high resistance levels to the pathogen [18,19,20]. Some reports indicated that the soybean isoflavones genistin (genistein 7-*O*-glucoside), which is the glycosylated form of genistein, and Biochanin A showed antibacterial activity against *Xcg* [21,22], and the application of the isoflavone genistein was reported to induce disease resistance in soybean plants to *Xcg* by promoting the accumulation of endogenous genistin [22]. Various natural compounds have been screened for bacterial pustule disease management. For example, *Lysobacter antibioticus*-produced *p*-aminobenzoic acid (pABA) altered *Xcg* membrane integrity by modifying the lipopolysaccharide (LPS) profile [23], whereas colloidal chitosan was reported to inhibit *Xcg* growth [24].

Dipicolinic acid (DPA; 2,6-pyridinedicarboxylic acid) is a main component of *Bacillus*, *Geobacillus*, and *Clostridium* spores [25]. DPA plays a key role in the resistance of bacterial spores to harsh environmental conditions [26,27]. DPA is released during spore germination [28,29] and exhibits a broad spectrum of antifungal activity against a wide range of plant fungal pathogens, showing an antifungal mode of action based on chitin biosynthesis inhibition [30]. DPA has been applied for the management of *Valsa pyri* in pear tree trunks [30], *Ceratocystis fimbriata* in sweet potato tubers [31], and *Epicoccum sorghinum* in maize leaves [32], achieving moderate to high control efficacies. Additionally, DPA was reported to induce disease resistance in kiwifruit to *Botrytis cinerea* by inducing the accumulation of antifungal phenolics [33]. A recent report indicated that DPA is also a key component in the spores of the fungus *Paecilomyces* [34]. DPA has been found in the culture medium of various *Paecilomyces* biocontrol strains, playing a key role in their antifungal and insecticidal activities [35,36,37]. DPA was also identified in the fungus *Simplicillium chinense*, showing activity against the root-knot nematode *Meloidogyne incognita* [38].

A few reports have indicated that DPA shows antibacterial activity against various Gram-negative human pathogenic bacteria, such as *Proteus vulgaris*, *Pseudomonas aeruginosa*, *Escherichia coli*, and *Staphylococcus aureus* [39,40]. DPA shows strong chelating properties and has been screened as a metallo-β-lactamase inhibitor due to its ability to remove zinc(II) from the enzyme active site [41]. DPA metal complexes formed with chromium(III), nickel(II), and copper(II) have also been evaluated as antibacterial agents [42,43,44,45]. DPA is inexpensive, and no toxicity to humans has been reported. Although DPA has been reported to show antibacterial activity against some Gram-negative human pathogens, its activity against plant-pathogenic bacteria, particularly *Xanthomonas* species, and its associated mechanisms of action have not been investigated. Various chelating agents, such as pyoverdines [46] and hydroxamate siderophores [47], which are able to coordinate iron, have been screened against *Xanthomonas*. This suggests that DPA, which can also coordinate to iron [48], could exhibit antibacterial activity against *Xanthomonas*.

As indicated above, DPA is a common component of *Bacillus* spores and is present in *Bacillus* culture medium. Numerous *Bacillus* strains have been screened as potential biocontrol agents, and various *Bacillus* strains have been commercialized [49,50]. Although the application of *Bacillus* biocontrol agents for the management of plant fungal pathogens is by far the most common, some commercial biocontrol agents based on *Bacillus* strains, such as *Bacillus amyloliquefaciens* F727 (Stargus^®^, Marrone Bio Innovations), *Bacillus subtilis* AFS032321 (Theia^®^, AgBiome Innovations), *B. subtilis* IAB/BS03 (Aviv^®^, Seipasa), and *B. subtilis* QST 713 (Serenade MAX^®^, Bayer), are recommended for the management of *Xanthomonas* blights, among other bacterial diseases [51]. Thus, the study of the antibacterial properties of DPA may help to understand a new antibacterial metabolite from *Bacillus* that has gone unnoticed to date.

This study aimed to examine the antibacterial mechanism of DPA and its potential antibacterial activity against various *Xanthomonas* pathovars, addressing a significant knowledge gap in the field. This study demonstrates that DPA shows antibacterial activity against *Xcg* and reveals a novel strategy for the management of soybean bacterial pustule disease.

## 2. Materials and Methods

### 2.1. Chemicals and Instruments

DPA (analytical grade; CAS: 499-83-2) was purchased from Macklin (99% purity; product number: P816098; China) and used in the experiments. Detailed information of all the reagents used in this study can be found in Supplementary Material Table S1.

Absorbance was measured using a 2300 EnSpire microplate reader (PerkinElmer, Waltham, MA, USA). Quantitative real-time polymerase chain reaction (qRT-PCR) analysis was carried out using a 7500 Real-Time PCR system (Applied Biosystems, Foster City, CA, USA). Crystal violet-stained *Xcg* cells were observed using a Leica DM2500 microscope (Wetzlar, Germany), while biofilm formation was analyzed using an Axio Observer 7 confocal microscope (Zeiss, Oberkochen, Germany). Scanning electron microscopy (SEM) images were collected using a Gemini 300 instrument (Zeiss, Oberkochen, Germany). Transmission electron microscopy (TEM) images were collected using an HT7800 instrument (Hitachi, Tokyo, Japan), while ultra-thin sections were prepared using a Leica EMUC7 ultramicrotome (Leica, Wetzlar, Germany).

### 2.2. Bacterial Strains and Culture Conditions

The representative strains *Xanthomonas campestris* pv. *campestris* FE58, *Xcg* ICMP5732, *Xanthomonas oryzae* pv. *oryzae* PXO99, *Xanthomonas oryzae* pv. *oryzicola* RS105, and *B. subtilis* 168, which have been widely used in genetic studies [52,53,54,55], were used in the experiments. To understand the role of DPA in the antibacterial activity of the biocontrol agent *B. subtilis*, *B. subtilis* 168 and the mutant Δ*spoVF*, obtained after deletion of the *spoVF* operon (DPA synthase gene), were used in the assay [56]. Similarly, to understand the role of DPA in the antibacterial activity of *Paecilomyces*, *P. maximus* NJC01 and mutant Δ*pmdpa*, obtained after deletion of the *pmdpa* gene (DPA synthase gene), were used in the assay [34]. The bacterial strains were maintained on Luria–Bertani (LB) semisolid medium (10 g/L tryptone, 5 g/L yeast extract, 10 g/L sodium chloride, and 15 g/L agar; pH adjusted to 7.0) at 28 °C and grown in LB broth (LB medium without agar) with shaking at 28 °C and 200 rpm. The fungal strains were maintained on potato-dextrose agar (PDA) medium (200 g peeled potatoes, 20 g glucose, and 15 g agar in 1 L double-distilled H_2_O (ddH_2_O)), and grown in potato-dextrose broth (PDB; PDA medium without agar) with shaking at 28 °C and 200 rpm.

Plant experiments were carried out using ‘ZH13’ soybean plants, which were grown under sterilized conditions in a growth chamber at 25 °C, 70% relative humidity, and 12 h of light per day.

### 2.3. Antibacterial Screening

*X. campestris* pv. *campestris* FE58, *Xcg* ICMP5732, *X. oryzae* pv. *oryzae* PXO99, and *X. oryzae* pv. *oryzicola* RS105 were used in the assay to understand the inhibitory activity of DPA on different *Xanthomonas* pathovars. Bacterial strains were grown in 10 mL LB medium with shaking at 28 °C and 200 rpm to OD_600_ = 1.0. Cells were collected after centrifugation at 6000× *g* and 4 °C for 10 min, washed twice with 5 mL sterilized ddH_2_O, and finally re-suspended in 10 mL sterilized ddH_2_O. Then, 500 μL of the resulting suspension was added to 10 mL LB medium containing 5, 10, 20, 50, 100, 200, and 500 μg/mL DPA. The cultures were then shaken at 28 °C and 200 rpm for 12 h. A control experiment was carried out using *B. subtilis* 168 [31]. This strain was used as a control to determine whether DPA can inhibit the growth of *B. subtilis*. Control experiments were carried out by evaluating the antibacterial activity of the chemical bactericides copper(II) chloride, kanamycin, streptomycin sulphate, and tetracycline against *Xcg*. These four bactericides have been previously screened for the management of *Xcg* [5]. The negative control experiment was carried out without antibacterial agents. Bacterial growth was monitored by measuring the OD_600_ value. Five replicates were performed for each treatment condition. Half-maximal effective concentration (EC_50_) values (the concentration that reduces cell growth by 50%) were calculated by the least-squares method using Prism 7.0 (GraphPad Software, San Diego, CA, USA). Minimum inhibitory concentration (MIC) values correspond to the concentration of antibacterial agent that completely inhibited *Xcg* growth. Given that DPA demonstrated significant antibacterial activity solely against *Xcg* and not the other pathovars, *Xcg* was used for the subsequent experiments. The MIC value was confirmed by treating *Xcg* cells with 500 μg/mL DPA in 10 mL LB medium for 24 h. Then, 100 μL of the medium were spread onto LB agar plates. No colony-forming unit (CFU) was observed on the plates.

The inhibitory effects of DPA on *Xcg* growth were also analyzed at different time points. *Xcg* was cultured in LB broth containing 0, 10, 20, 50, 100, 200, and 500 μg/mL DPA at 28 °C and 200 rpm. *Xcg* growth was monitored by measuring the OD_600_ values at 0, 1, 2, 4, 8, 12, and 24 h. Five replicates were performed for each treatment condition and time point.

### 2.4. Determination of Swimming Motility, Extracellular Protease Activity, and Biofilm Formation

Swimming motility, extracellular protease activity, and biofilm formation were determined as previously reported by our research group [23]. Briefly, *Xcg* was grown in 5 mL LB medium to OD_600_ = 1.0. Swimming motility was determined by adding 3 μL of bacterial culture medium on the surface of a Petri dish containing LB agar (the concentration of agar was 15 g/L) medium and 0, 50, 100, 200, and 500 μg/mL DPA. When culturing, the bacterial colonies were on the upper side of the plates. The diameter of the colonies was measured after static incubation at 28 °C and 70% relative humidity for 48 h. The diameter of colonies in the treatment groups was compared with that in the control group, which was carried out in the absence of DPA. Five replicates were carried out for each treatment condition.

Extracellular protease activity was assessed as previously reported by our research group by analyzing the hydrolytic zone diameter caused by *Xcg* in a medium containing skim milk powder [23]. Three microliters of bacterial culture medium (OD_600_ = 1.0) were spotted on LB-agar plates containing 1% skim milk powder (≥34% protein of milk solid non-fat; product number: BS102-500g; Biosharp, Hefei, China) and 0, 50, 100, 200, and 500 μg/mL DPA. The protease activity was calculated according to the length of the hydrolytic zone around the bacterial colonies after incubation at 28 °C for 48 h. Five replicates were carried out for each treatment condition.

To test biofilm formation, 50 μL of bacterial culture was inoculated into 5 mL LB medium (in glass tubes with rubber stoppers) in the presence of 0, 50, 100, 200, and 500 μg/mL DPA. These cultures were incubated at 28 °C without shaking for 3 days. After removing the solution, 10% *w*/*v* crystal violet methanolic solution (6 mL) was added to the biofilm, and the resulting suspension was maintained at room temperature for 1 h. Then, the solution was removed, and the stained biofilms were washed twice with sterile ddH_2_O (3 mL) and dried at room temperature for 1 h. Finally, 3 mL of a 50:40:10 ddH_2_O/methanol/glacial acetic acid mixture was added to the glass tubes to dissolve the crystal violet stain. The dissolved crystal violet was quantified at 575 nm. Each treatment condition was repeated five times. Biofilm formation was also assessed using microscopy as indicated in Section 2.5.

### 2.5. Biofilm Observations via Laser Scanning Confocal Microscopy (LSCM)

*Xcg* biofilm was prepared by following the conditions described in Section 2.4. *Xcg* biofilm formation was induced in LB medium containing 50, 100, 200, and 500 μg/mL DPA at 28 °C for 3 days without shaking. The control experiment was carried out without DPA. Biofilm was collected by centrifuging at 6000× *g* and 4 °C for 5 min. After washing the biofilm twice with 200 μL ddH_2_O, it was treated with calcofluor-white staining solution (50 μL) for 1 min [57]. The dye was eliminated by washing twice with 200 μL ddH_2_O, and the biofilm was examined with a confocal microscope at 405 nm (blue color). The integrated intensity was recorded for ten different samples from each treatment group.

### 2.6. Determination of Reducing Sugar Content in Xcg Biofilm

*Xcg* biofilm was prepared following the conditions described in Section 2.4. *Xcg* biofilm formation was induced in LB medium containing 50, 100, 200, and 500 μg/mL DPA at 28 °C for 3 days without shaking. The control experiment was carried out without DPA. The biofilm was collected and treated with 100 μL 6 M HCl and 150 μL ddH_2_O. Then, the resulting suspension was heated at 100 °C for 30 min. The total content of reducing sugars in the solution was measured using the 3,5-dinitrosalicylic acid (DNS) method [58]. The principle of this method relies on the reaction between DNS and free reducing sugars to produce 3-amino-5-nitrosalicylic acid, which shows absorbance at 540 nm. A linear calibration was established using commercial glucose. Five replicates were carried out for each treatment condition.

### 2.7. Microscope Observations of Xcg Cells After Crystal Violet Staining and Assessment of DPA Bactericidal Activity

To observe *Xcg* cells after crystal violet staining, *Xcg* was grown in 10 mL LB medium containing 100 μg/mL DPA to OD_600_ = 1.0 [23]. The control experiment was carried out without DPA. Cells were collected via centrifugation at 8000× *g* and 4 °C for 8 min. The harvested cells were resuspended in ddH_2_O to OD_600_ = 0.2. Then, 20 μL of cell suspension was added to a microscope slide, and the solution was evaporated at 50 °C. The cells were stained with 5 μL crystal violet solution (2 g crystal violet in 20 mL of 95% *v*/*v* ethanol and 80 mL of 1% *w*/*v* ammonium acetate aqueous solution) for 2 min and then washed twice with 5 μL ddH_2_O. Afterwards, *Xcg* cells were covered with 5 μL iodine solution (5 g iodine and 10 g potassium iodide in 100 mL ddH_2_O) for 2 min and washed twice with 5 μL ddH_2_O. Cells were then dyed with 5 μL safranine solution (2.5 g safranine in 100 mL of 95% *v*/*v* ethanol and 80 mL ddH_2_O) for 2 min and washed twice with 5 μL ddH_2_O. The cells were then observed using a Leica DM2500 microscope at ×40 and ×100 magnifications.

To assess the lethal effects of DPA on *Xcg* cells, the cells were shaken at 28 °C and 200 rpm in 10 mL LB medium for 5 h in the presence of 0, 50, 100, and 500 μg/mL DPA. After this exposure, a 50 μL aliquot was transferred into 10 mL of fresh LB medium (without DPA), and the resulting suspensions were shaken at 28 °C and 200 rpm for 12 h. Bacterial growth was monitored by measuring OD_600_. The inability of cells to grow after DPA exposure indicates lethal (bactericidal) effects. Five replicates were performed for each treatment condition. The effects of DPA on the cell morphology were observed using a Ti2-E-AXR microscope (Nikon, Tokyo, Japan).

### 2.8. SEM Imaging

To observe *Xcg* cells by SEM, *Xcg* was grown in 10 mL LB medium containing 100 μg/mL DPA to OD_600_ = 1.0. The control experiment was carried out without DPA. Cells were harvested by centrifuging at 8000× *g* and 4 °C for 8 min and immobilized using 4% *v*/*v* *p*-formaldehyde aqueous solution. Then, the samples were dehydrated using 65%, 75%, 85%, 95%, and 100% (twice) *v*/*v* ethanol (each dehydration condition was carried out for 8 min) [23]. Dried samples were placed on the sample holder. After coating with gold, the images were obtained with 20 kV acceleration voltage. The size of one hundred cells was measured to calculate the average cell length and width.

### 2.9. TEM Imaging

To observe *Xcg* cells by TEM, *Xcg* was grown in 10 mL LB medium containing 100 μg/mL DPA to OD_600_ = 1.0. The control experiment was carried out without DPA. Cells were harvested by centrifuging at 8000× *g* and 4 °C for 8 min. After immobilization using 2.5% *v*/*v* glutaraldehyde aqueous solution, the samples were dehydrated with 50%, 70%, and 90% (twice) *v*/*v* ethanol at 4 °C (each dehydration condition was carried out for 15 min). After dehydration, the samples were dried at 37 °C for 12 h, at 45 °C for 48 h, and at 60 °C for 48 h [59]. Ultra-thin cuts of 70 nm were made in the block and contrasted with 2% uranyl acetate for 15 min and lead citrate for 20 min. Images were collected at ×20,000 magnification and a voltage of 80 kV.

### 2.10. qRT-PCR Analysis

*Xcg* was grown in 100 mL LB medium containing 100 μg/mL DPA to OD_600_ = 1.0. The control experiment was carried out without DPA. The cells were collected by centrifuging at 6000× *g* and 4 °C for 8 min. RNA extraction was carried out as previously reported, with minor modifications [21]. After extracting total RNA using TRIzol reagent (Thermo Fisher Scientific, Waltham, MA, USA), cDNA was synthesized with the HiScript III RT SuperMix for qPCR (+gDNA wiper) Kit (Vazyme, Nanjing, China). qRT-PCR was performed using the Taq Pro Universal SYBR qPCR Master Mix Kit (Vazyme, Nanjing, China). *16S rRNA* was used as the reference gene. The relative gene expression was calculated using the 2^−ΔΔCT^ method. Given that SEM and TEM images suggested that DPA was causing severe effects on *Xcg* membrane integrity, the expression of genes associated with membrane integrity was measured. The selected genes were chosen based on their established roles in Gram-negative bacterial membrane integrity and efflux systems. *cirA*, *czcA*, *czcB*, and *emrE* encode outer membrane proteins involved in heavy metal resistance and osmotic balance maintenance [9,60,61]. *tolC* is a key component of multidrug efflux pumps and contributes to outer membrane stability [62]. *kefB* is involved in potassium ion transport and pH homeostasis [63]. Genes *342RT*, *1501RT*, and *2689RT* encode resistance-nodulation-cell-division (RND) efflux system membrane fusion-like proteins [64]. In addition, the expression of various cell division genes, including *4495RT*, *ftsA*, *ftsL*, *ftsZ*, *minD*, *zapA*, and *zipA*, and pathogenesis-related genes, including *1578RT*, *rpfE*, and *yapH*, was examined [23]. The primers used in the qRT-PCR assay are detailed in Supplementary Material Table S2. Five replicates were carried out for each treatment condition.

### 2.11. Assessment of the Role of DPA in the Antibacterial Activity of Cell-Free Supernatants from B. subtilis 168 and P. maximus NJC01

*B. subtilis* 168 and the mutant Δ*spoVF* [56], which does not synthesize DPA, were grown in 200 mL LB medium with shaking at 28 °C and 200 rpm to OD_600_ = 1.0. *P. maximus* NJC01 and the mutant Δ*pmdpa* [34], which does not synthesize DPA, were grown in 200 mL PDB medium with shaking at 28 °C and 200 rpm to an OD_600_ of 1.0. Cell suspensions were centrifuged at 8000× *g* and 4 °C for 10 min, and the cell-free supernatants were collected. The cell-free supernatants from the four strains were mixed with LB at 1%, 2%, 5%, 10%, and 20% *v*/*v* concentrations (total volume = 1 mL). *Xcg* was grown in 10 mL LB medium with shaking at 28 °C and 200 rpm to OD600 = 1.0. Cells were collected by centrifugation at 6000× *g* and 4 °C for 10 min, washed twice with 5 mL sterilized ddH_2_O, and finally resuspended in 10 mL sterilized ddH_2_O (OD_600_ = 1.0). Five microliters of the resulting suspension were added to each cell-free supernatant-LB mixture. The control experiment was carried out in the absence of supernatant. The cultures were shaken at 28 °C and 200 rpm for 5 h. The antibacterial activity of the cell-free supernatant was calculated by measuring the OD_600_ value of the cultures. Five replicates were carried out for each treatment condition.

### 2.12. In Vivo Assay for the Management of Bacterial Pustule Disease in Soybean

The efficacy of DPA for the management of bacterial pustule disease was screened using preventive and curative assays [23]. Three-week-old soybean plants, with three trifoliate leaves each, approximately 50–60 cm tall, were used for the inoculation of the pathogen. *Xcg* was grown in 100 mL LB medium to OD_600_ = 1.5. Cells were harvested by centrifuging at 6000× *g* and 4 °C for 10 min, washed twice with sterilized ddH_2_O (10 mL), and resuspended in sterilized ddH_2_O to OD_600_ = 0.8.

In the preventive assay, 100 mL aqueous solutions (pH 6) containing 50, 100, 200, and 500 μg/mL DPA were sprayed on the soybean plants (100 mL DPA solution for 25 soybean plants). After drying at room temperature for 2 h, *Xcg* (20 mL aqueous solution containing bacterial cells; OD_600_ = 0.8) was sprayed onto the upper surface of the leaves of 25 soybean plants.

In the curative assay, *Xcg* (20 mL aqueous solution containing bacterial cells; OD_600_ = 0.8) was sprayed onto the upper surface of the leaves of 25 soybean plants, and the soybean plants were stored at 25 °C and 70% relative humidity for 10 h, observing weak bacterial pustule disease symptoms (yellow lesions) on the leaves. Then, aqueous solutions containing 50, 100, 200, and 500 μg/mL DPA (100 mL, pH 6) were sprayed (100 mL DPA solution for 25 soybean plants) on the soybean leaves.

Copper(II) chloride (100 mL; 50 μg/mL) (only active ingredient; product number: C805298; Macklin, Shanghai, China) was applied as a positive control, whereas sterilized ddH_2_O (100 mL) was sprayed in the negative control experiment. The number of lesions per leaf was monitored at 1, 2, 3, and 4 days after inoculation of the pathogen. Two repetitions were carried out. The number of lesions in approximately 150 leaves was measured and pooled to calculate the mean and standard deviation.

### 2.13. Preparation of Leaf Extracts

Soybean leaves were inoculated with *Xcg* using the preventive and curative procedures described in Section 2.12. Soybean leaf extracts were prepared following a standard procedure [22]. Briefly, soybean leaves were collected at days 0, 1, 2, 3, and 4 after pathogen inoculation and ground into powder using liquid nitrogen. One milligram of the powder was suspended in 1 mL of 80% *v*/*v* aqueous methanol and sonicated at room temperature for 20 min. The suspension was centrifuged at 6000× *g* and 4 °C for 10 min, and the supernatant was collected and stored at −80 °C until use. Leaf extracts were used to measure total phenolic content (TPC) (Section 2.13) and genistin concentration (Section 2.14).

### 2.14. Measurement of TPC in Xcg-Infected Soybean Leaves

TPC was measured following a previously reported procedure [22]. Soybean leaf extract (20 μL) was mixed with 40 μL of 10% *v*/*v* Folin–Ciocalteu reagent. The resulting suspension was kept in the dark for 5 min. Then, 140 μL of 7% *w*/*v* sodium carbonate was added. After 90 min in darkness, the absorbance at 750 nm was measured. The calibration was performed using gallic acid, and the results were expressed according to the mg gallic acid equivalents per gram of leaf (mgGAE/g). Five replicates were carried out for each treatment condition and time point.

### 2.15. Measurement of Genistin Level in Xcg-Infected Soybean Leaves

Soybean leaf extracts obtained on day 3 after pathogen inoculation were used for the detection of genistin. Acetonitrile and ddH_2_O were used as the eluents. A linear gradient from 13% to 30% acetonitrile (70 min) was used to elute soybean isoflavones, with a flow rate of 1.0 mL/min [22]. Absorbance, column temperature, and injection volume were set at 260 nm, 35 °C, and 10 μL, respectively [22]. The peak corresponding to genistin was observed at 19.1 min retention time. The calibration was performed using commercial genistin. Five replicates were carried out for each treatment condition.

### 2.16. Evaluation of DPA Toxicity in the Zebrafish Model

The developmental toxicity of DPA was evaluated using zebrafish (*Danio rerio*) embryos following a previously reported protocol [65] with minor modifications. Zebrafish embryos were obtained from the Zebrafish Center of Nantong University and maintained under standard conditions [66]. Healthy embryos at 4 h post-fertilization (hpf) were randomly distributed into 24-well plates (20 embryos per well) containing 5 mL of E3 medium (5 mM NaCl, 0.17 mM KCl, 0.33 mM CaCl_2_, and 0.33 mM MgSO_4_). Embryos were exposed to DPA at concentrations of 0, 5, 10, 20, 50, 100, 200, and 500 μg/mL. The control group was incubated in E3 medium without DPA. All groups were maintained at 28 °C under a 14 h light/10 h dark cycle for 4 days (until 4 days post-fertilization, dpf). The exposure medium was renewed daily. Three independent replicates were performed, each with 20 embryos per treatment (n = 60 embryos per treatment condition).

At 4 dpf, mortality was recorded, and surviving larvae were used for further analyses. Heart rate was measured by counting the number of heartbeats within 30 s using a Stemi 508 stereomicroscope (Zeiss, Oberkochen, Germany) and expressed as beats per minute (bpm). For body length measurement, larvae were anesthetized with 0.02% tricaine (MS-222), placed on a glass slide, and photographed using an MVX10 macroscopic zoom stereomicroscope (Olympus, Tokyo, Japan). Body length (from the tip of the head to the end of the tail) was measured using the cellSens Imaging software version 1.16 (Olympus, okyo, Japan). Animal experimentation was approved by the Administration Committee of Experimental Animals, Jiangsu Province, China (Approval Number: 20200711-001).

### 2.17. Statistical Analysis

DPA antibacterial activity over time, swimming motility, protease activity, biofilm formation, content of reducing sugars, in vivo efficacy, TPC, and genistin level variables were analyzed using ANOVA followed by post hoc Tukey’s test (*p* < 0.05) (SPSS software; IBM version 27.0.1.0, Armonk, NY, USA). qRT-PCR, antibacterial activity of the cell-free supernatants, mortality rate, and heart rate per minute variables were submitted to Student’s *t* test by comparing the treatment groups with the control groups. ANOVA assumptions for normality and homogeneity were tested. Five replicates were carried out to measure bacterial growth, swimming motility, extracellular protease activity, biofilm formation, reducing sugar content, cell-free supernatant antibacterial activity, TPC, and genistin concentration (n = 5). The qRT-PCR assay also consisted of five biological replicates (n = 5). DPA efficacy in soybean leaves was measured in approximately 75 leaves for each treatment condition, with two repetitions (n = 150). DPA toxicity in the zebrafish model was evaluated using 60 embryos (n = 60). Error bars indicate standard deviation.

## 3. Results

### 3.1. DPA Inhibits Xcg Growth

DPA efficiently inhibited the growth of *Xcg* ICMP5732. However, DPA only showed weak antibacterial activity against *X. campestris* pv. *campestris* FE58, *X. oryzae* pv. *oryzae* PXO99, and *X. oryzae* pv. *oryzicola* RS105 (Table 1). The EC_50_ and MIC values of DPA against *Xcg* were 53.2 ± 3.5 and 500 μg/mL, respectively. DPA did not significantly affect the growth of *B. subtilis* 168. Copper(II) chloride exhibited lower antibacterial activity against *Xcg* than DPA, with an EC_50_ of 123.3 ± 5.6 μg/mL (Table 1). In contrast, the organic bactericides kanamycin, streptomycin sulphate, and tetracycline showed higher antibacterial activity against *Xcg* than DPA. These three organic bactericides had EC_50_ values equal to or lower than 11.2 μg/mL.

**Figure 1:**
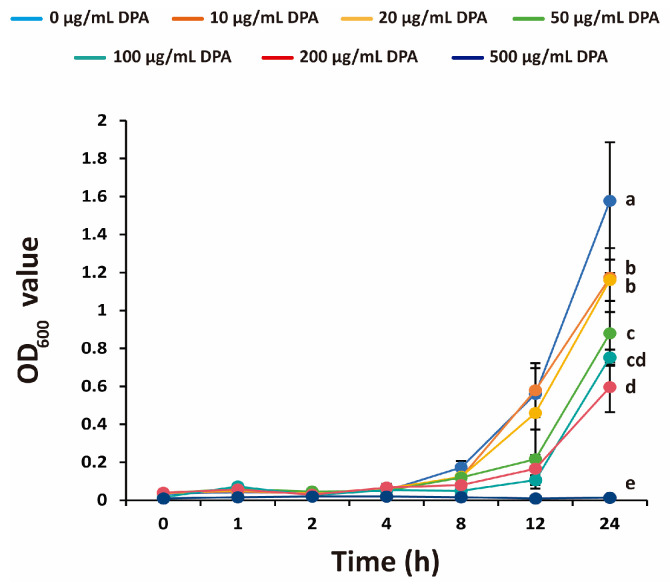
Inhibitory effects of DPA on *Xanthomonas citri* pv. *glycines* (*Xcg*) growth at different concentrations and time points. The control experiment was carried out without DPA. Different letters indicate that the means were statistically different after 24 h of culture (*p* ≤ 0.05).

The antibacterial activity of DPA against *Xcg* over time was also examined. Interestingly, 500 μg/mL DPA completely inhibited *Xcg* for 24 h (Figure 1). After 24 h of culture, DPA at 100 and 200 μg/mL reduced the OD_600_ value by 52.2% and 62.2%, respectively, compared to the control group. This is the first experimental evidence confirming the antibacterial activity of DPA against a plant bacterial pathogen.

### 3.2. DPA Inhibits Xcg Swimming Motility and Extracellular Protease Activity

**Figure 2:**
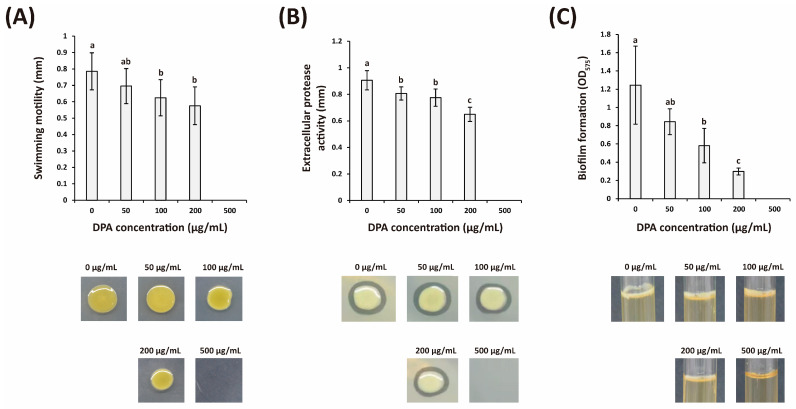
Effects of DPA on (**A**) swimming motility, (**B**) protease activity, and (**C**) biofilm formation of *Xanthomonas citri* pv. *glycines* (*Xcg*). Swimming motility, protease activity, and biofilm formation were measured as previously reported [23]. Different lower-case letters indicate that the means were statistically different (*p* ≤ 0.05).

DPA at 100 and 200 μg/mL significantly reduced *Xcg* swimming motility and extracellular protease activity. When treating with 100 and 200 μg/mL DPA, *Xcg* colony diameter was reduced by 20.5% and 26.7%, respectively (Figure 2A). In the assay using skim milk powder, application of 100 and 200 μg/mL DPA reduced the length of the hydrolytic zone by 14.5% and 28.3%, respectively (Figure 2B).

### 3.3. DPA Inhibits Xcg Biofilm Formation and Decreases Biofilm Reducing Sugar Content

The application of 100 and 200 μg/mL DPA reduced the absorbance at 575 nm after biofilm staining by 53.2% and 76.0%, respectively (Figure 2C), confirming that DPA can inhibit biofilm formation. The effects caused by DPA on biofilm formation were more significant than those observed on swimming motility and extracellular protease activity.

**Figure 3:**
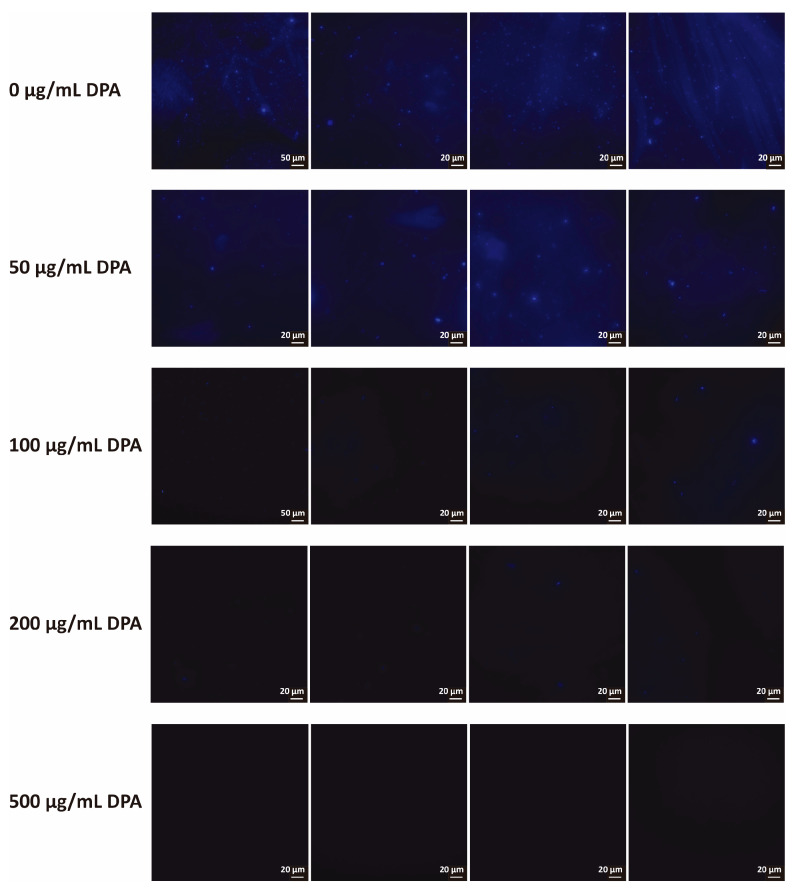
Confocal microscopy characterization of *Xanthomonas citri* pv. *glycines* (*Xcg*) biofilm after treatment with DPA. Biofilms were stained with calcofluor white. The control experiment was carried out without DPA. Bar = 20 or 50 μm.

Confocal microscopy observations showed that *Xcg* biofilm was distributed in the absence of DPA (Figure 3). However, after treatment with DPA, *Xcg* biofilm was less intense and highly dispersed. Almost no structures were observed after application of 200 and 500 μg/mL DPA. The integrated intensity recorded for the confocal images was 395,081.3333 ± 402,162.0831, 95,703.6667 ± 39,970.5059, 26,398.6667 ± 10,195.9877, 6120.3333 ± 1899.9467, and 0.0000 ± 0.0000 after application of 0, 50, 100, 200, and 500 μg/mL DPA, respectively.

**Figure 4:**
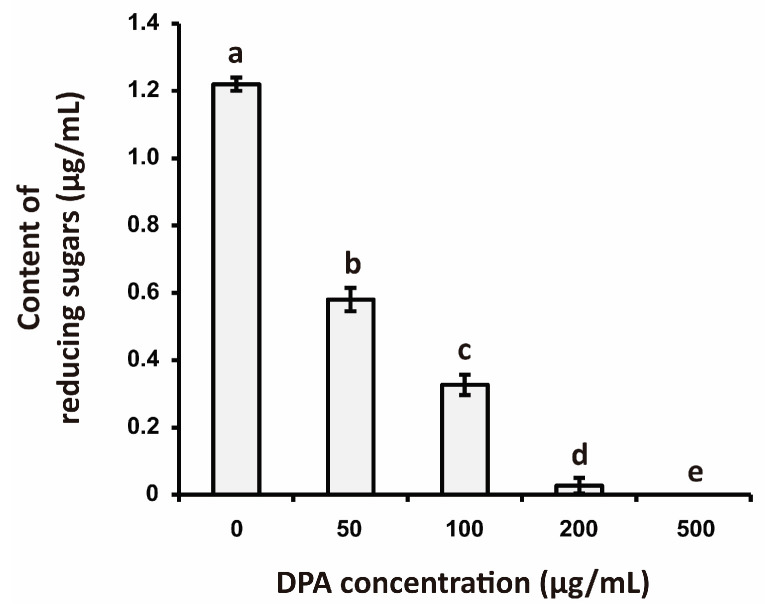
Effects of DPA on the reducing sugar content of *Xanthomonas citri* pv. *glycines* (*Xcg*) biofilm. The total content of reducing sugars was measured using the 3,5-dinitrosalicylic acid (DNS) method [58]. The results are expressed as content of reducing sugars (μg/mL glucose equivalents). Different lower-case letters indicate that the means were statistically different (*p* ≤ 0.05).

The results showed that the reducing sugar content in *Xcg* biofilm after application of 100 μg/mL DPA was 73.2% lower than that observed in the control experiment, while 200 μg/mL DPA decreased reducing sugar content in *Xcg* biofilm by 97.8% (Figure 4).

### 3.4. DPA Alters Xcg Morphology and Induces Cell Lysis

Microscope observations after crystal violet staining indicated that 100 μg/mL DPA induced *Xcg* cell lysis (Supplementary Material Figure S1). The cell integrity remained intact in the absence of DPA, and numerous bacterial consortia were observed. Instead, the number of cells was significantly reduced after treatment with DPA. *Xcg* cell degradation resulted in amorphous residues. Assessment of the bactericidal activity of DPA revealed that treatment with 50, 100, and 500 μg/mL DPA for 5 h resulted in cells with irregular morphologies in a concentration-dependent manner (Supplementary Material Figure S2). After treatment with 500 μg/mL DPA for 5 h, cell lysis was detected in a large proportion of cells. When DPA-treated cells were re-cultured in LB medium, the cultures reached OD_600_ values of 0.44 ± 0.07 (0 μg/mL DPA), 0.36 ± 0.02 (50 μg/mL DPA), 0.37 ± 0.02 (100 μg/mL DPA), and 0.14 ± 0.04 (500 μg/mL DPA). These results suggest that the effects of DPA on the *Xcg* membrane are reversible unless cell lysis occurs. Furthermore, the observation that DPA-treated *Xcg* cells can grow after re-culturing indicates that the effects of DPA on the cell membrane are not a consequence of cell death.

**Figure 5:**
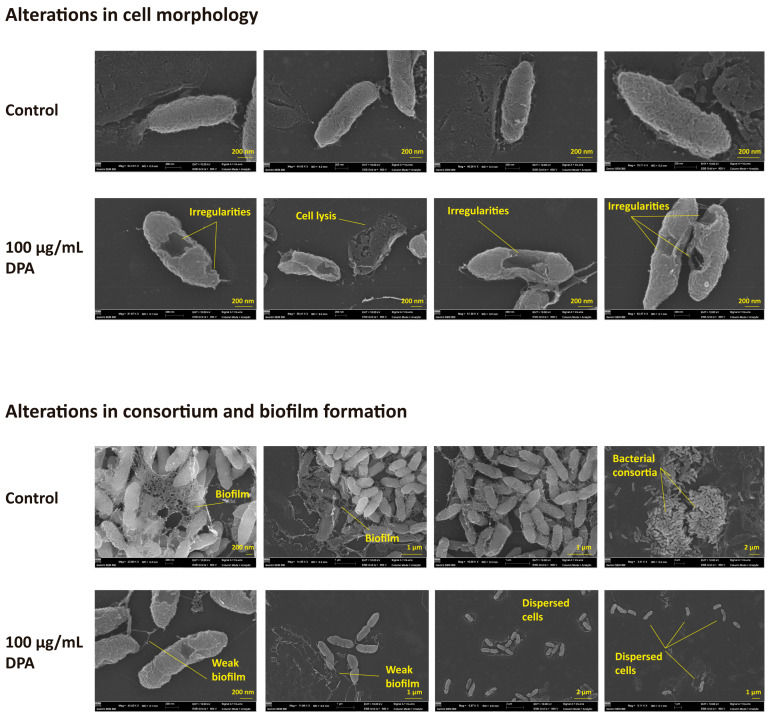
Scanning electron microscopy (SEM) images of *Xanthomonas citri* pv. *glycines* (*Xcg*) cells showing membrane damage and biofilm disruption. The bacterial cells were treated with 100 μg/mL DPA. The control experiment was carried out without DPA.

Consistently, SEM images showed that DPA caused relevant irregularities in the *Xcg* membrane, resulting in cell lysis (Figure 5). Although the untreated *Xcg* cells formed consortia and spider web-like biofilm, DPA-treated cells were dispersed and did not produce biofilm. SEM was used to measure the ratio of broken cells, and *Xcg* cell length and width. The ratio of broken cells without DPA was 4.19% ± 0.80%. However, the ratio of broken cells after DPA treatment (100 μg/mL) was as high as 47.52% ± 4.20%. This indicates that DPA is inducing cell lysis (*p* < 0.05). After treatment with 100 μg/mL DPA, *Xcg* cells were 1.14 ± 0.15 μm in length and 0.22 ± 0.02 μm in width, whereas in the absence of DPA, *Xcg* cells were 1.33 ± 0.23 μm in length and 0.22 ± 0.02 μm in width. Thus, DPA significantly reduced *Xcg* cell length (*p* < 0.05). However, no difference was observed in the width of the untreated and treated cells.

**Figure 6:**
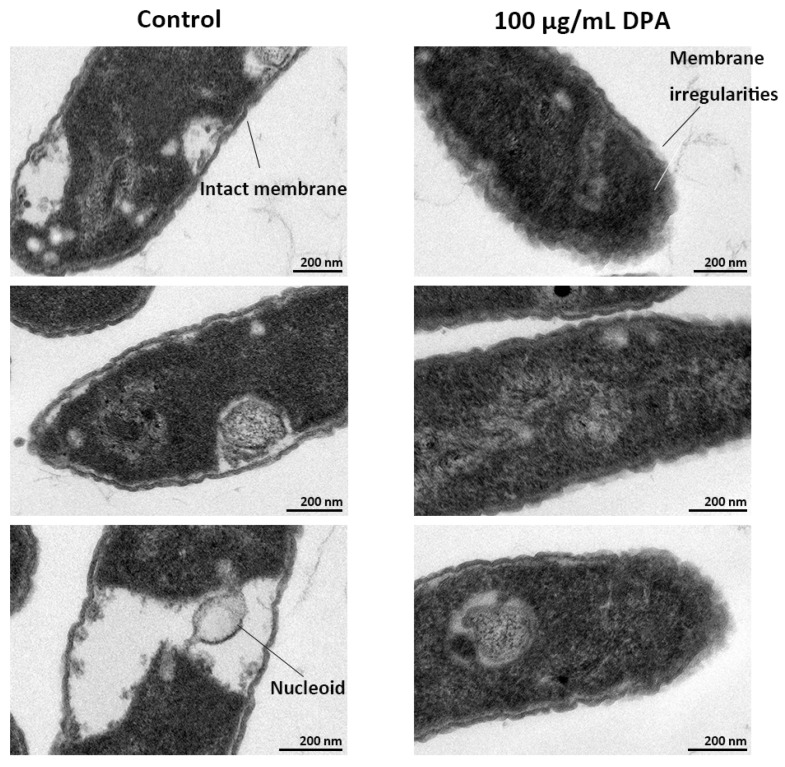
Transmission electron microscopy (TEM) observations of *Xanthomonas citri* pv. *glycines* (*Xcg*) cell morphology and internal structure. The bacterial cells were treated with 100 μg/mL DPA. The control experiment was carried out without DPA.

When using TEM, the untreated *Xcg* cells showed an intact cell membrane (Figure 6). White areas were detected inside the untreated cells, corresponding to the nucleoid [59]. Instead, the cell envelope of the DPA-treated cells was not well defined, confirming that DPA is causing irregularities in the cell membrane. The white areas corresponding to the nucleoid were not detected in the treated cells, suggesting that DPA is also causing alterations in the cytoplasm.

### 3.5. DPA Downregulates the Expression of Membrane Integrity-Related Genes

**Figure 7:**
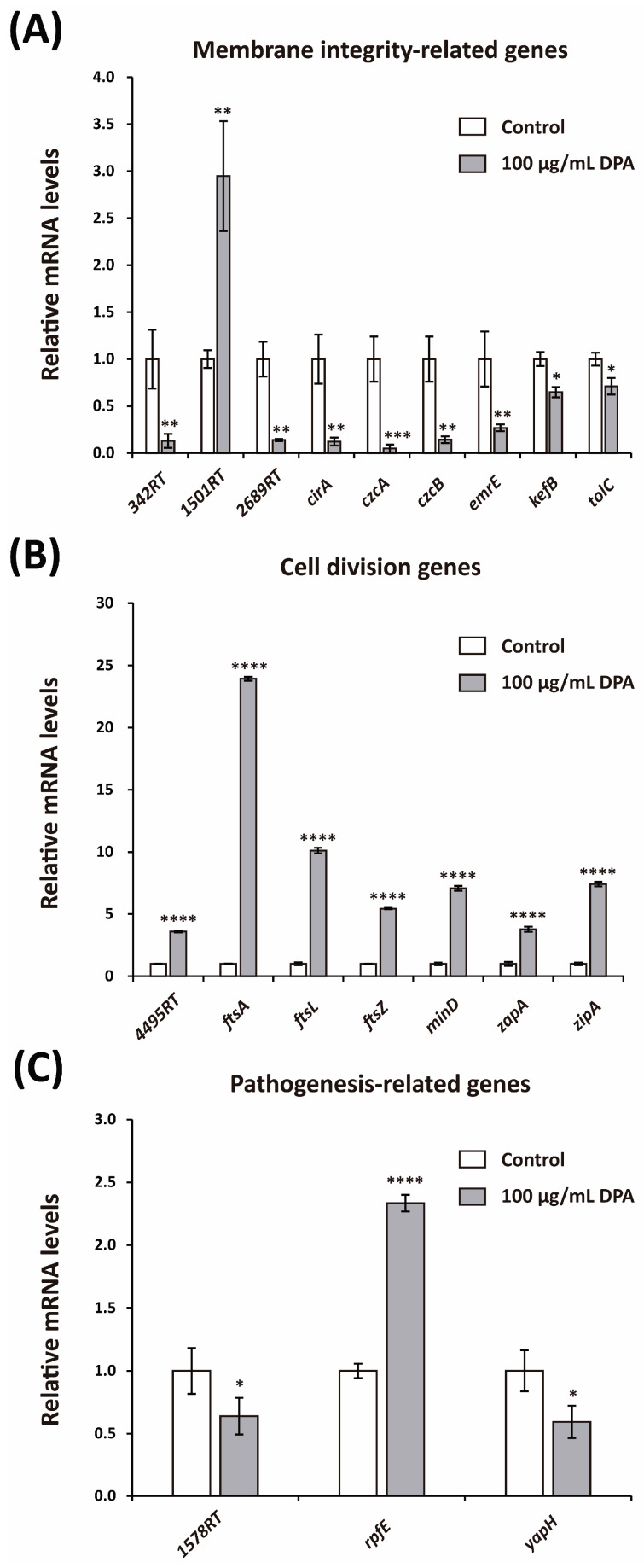
qRT-PCR analysis of relevant genes in DPA-treated *Xanthomonas citri* pv. *glycines* (*Xcg*) cells. (**A**) Expression of membrane integrity-related genes. (**B**) Expression of cell division genes. (**C**) Expression of pathogenesis-related genes. DPA was applied at 100 μg/mL concentration. The control experiment was carried out without DPA. The primers used in the qRT-PCR analysis are indicated in Supplementary Material Table S2. Significance levels: *, *p* ≤ 0.05; **, *p* ≤ 0.01; ***, *p* ≤ 0.001; ****, *p* ≤ 0.0001.

In agreement with the microscope observations, the mRNA levels of several key genes associated with membrane integrity were altered after DPA treatment (Figure 7A). Genes *342RT* and *2689RT* were downregulated 0.129- and 0.139-fold, respectively, after DPA treatment. Instead, the expression of *1501RT* was 2.947-fold higher after treatment with DPA. Several genes encoding outer membrane proteins (OMPs), including *cirA*, czcA, *czcB*, and *emrE*, were downregulated after DPA treatment. The mRNA levels of *cirA*, *czcA*, *czcB*, and *emrE* were 0.123-, 0.049-, 0.143-, and 0.269-fold lower, respectively, than those in the control group. Potassium efflux system gene *kefB* was 0.648-fold downregulated after treatment with DPA, whereas the outer membrane efflux gene *tolC* was 0.710-fold downregulated.

Cell division genes were upregulated after treatment with 100 μg/mL DPA (Figure 7B). Specifically, *4495RT*, *ftsA*, *ftsL*, *ftsZ*, *minD*, *zapA*, and *zipA* were 3.606-, 23.938-, 10.113-, 5.434-, 7.075-, 3.775-, and 7.406-fold upregulated, respectively, after treatment with DPA. This suggests that DPA accelerates *Xcg* cell division, which is not necessarily indicative of a pathogenic effect. The effects of DPA on pathogenesis-related genes did not show an obvious trend (Figure 7C). *1578RT* and *yapH* were 0.639- and 0.594-fold downregulated after DPA treatment. In contrast, *rpfE* was 2.334-fold upregulated.

### 3.6. DPA Plays a Key Role in the Antibacterial Activity of B. subtilis and P. maximus Cell-Free Supernatants

**Figure 8:**
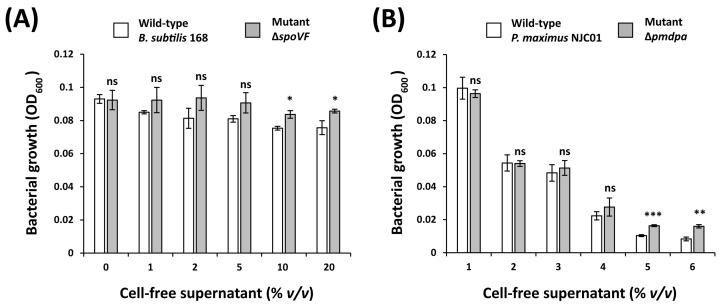
Role of DPA in the antibacterial activity of cell-free supernatants from the biocontrol agents *Bacillus subtilis* and *Paecilomyces maximus*. (**A**) Antibacterial activity of cell-free supernatants from wild-type *B. subtilis* 168 and the Δ*spoVF* mutant (DPA synthase-deficient). (**B**) Antibacterial activity of cell-free supernatants from wild-type *P. maximus* NJC01 and the Δ*pmdpa* mutant (DPA synthase-deficient). Wild-type strains exhibited higher antibacterial activity than their corresponding mutant strains, confirming that DPA plays a key role in the antibacterial activity of the cell-free supernatants from these biocontrol agents. Significance levels: *, *p* ≤ 0.05; **, *p* ≤ 0.01; ***, *p* ≤ 0.001; ns, no significance.

To investigate the role of DPA in the antibacterial activity of *B. subtilis* and *P. maximus*, the cell-free supernatants of wild-type *B. subtilis* 168 and the Δ*spoVF* mutant, as well as wild-type *P. maximus* NJC01 and the Δ*pmdpa* mutant, were tested against *Xcg* (Figure 8). At a concentration of 20% *v*/*v*, the wild-type *B. subtilis* supernatant reduced the OD_600_ of *Xcg* by 18.6% compared to the control, whereas the Δ*spoVF* mutant supernatant reduced it by only 7.2% at the same concentration. Similarly, the wild-type *P. maximus* supernatant at 20% *v*/*v* reduced *Xcg* OD_600_ by 91.6%, while the Δ*pmdpa* mutant supernatant reduced it by 83.4% under the same conditions. These results confirm that DPA contributes to the antibacterial activity of the biocontrol agents *B. subtilis* and *P. maximus*.

### 3.7. DPA Reduces Bacterial Pustule Disease Symptoms

**Figure 9:**
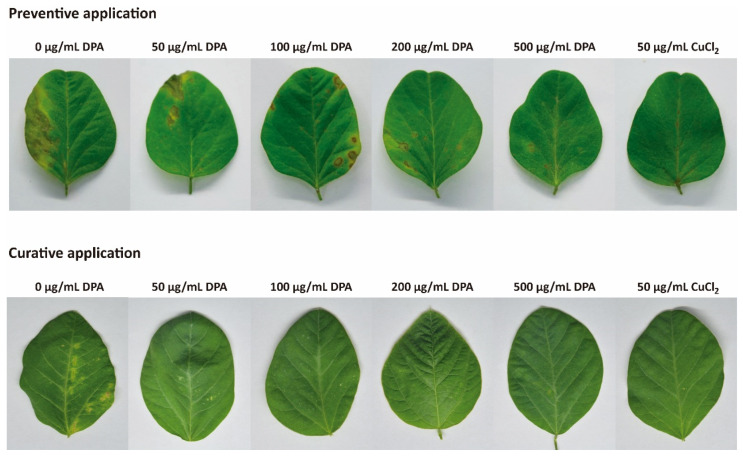
Bacterial pustule disease symptoms caused by *Xanthomonas citri* pv. *glycines* (*Xcg*) after treatment with DPA and copper(II) chloride. Antibacterial compounds were screened in preventive and curative applications. Sterilized ddH_2_O was sprayed in the control experiment.

DPA reduced bacterial pustule disease symptoms in soybean plants in a concentration-dependent manner (Figure 9 and Table 2). The higher the DPA concentration, the higher the inhibitory effect. Preventive application of 50, 100, 200, and 500 μg/mL DPA reduced the number of lesions by 26.9%, 51.2%, 64.1%, and 82.7% at 4 days post-inoculation, respectively, compared to the control group, which was carried out without DPA.

Similarly, curative application of 50, 100, 200, and 500 μg/mL DPA reduced the number of lesions by 28.1%, 32.2%, 66.5%, and 83.8% at 4 days post-inoculation, respectively, compared to the control group (Figure 9 and Table 2). It must be noted that 500 μg/mL DPA allowed the same preventive efficacy (82.7% inhibition) compared to 50 μg/mL copper(II) chloride.

### 3.8. DPA Enhances TPC and Genistin Level in Xcg-Infected Soybean Leaves

**Figure 10:**
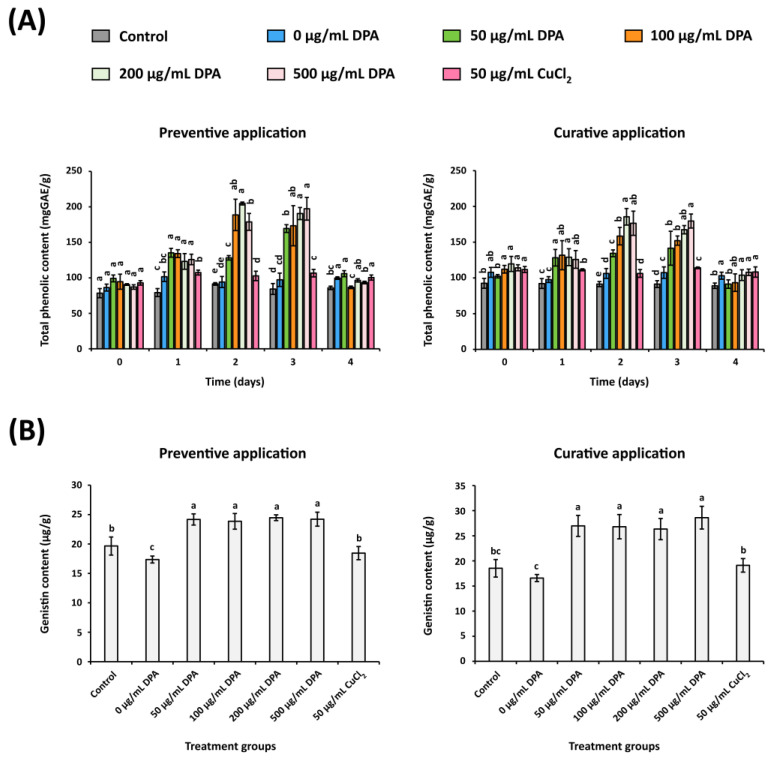
Effects of DPA and copper(II) chloride on the (**A**) total phenolic content (TPC) and (**B**) genistin level of *Xanthomonas citri* pv. *glycines* (*Xcg*)-infected soybean leaves. DPA was applied at 0, 50, 100, 200, and 500 μg/mL, while copper(II) chloride was applied at 50 μg/mL. The effects were analyzed in both preventive and curative applications. The control experiment was carried out without DPA, copper(II) chloride, and *Xcg*. Different lower-case letters indicate that the means were statistically different (*p* ≤ 0.05). In the TPC assay, each time point was considered a different statistical group. Abbreviations: GAE, gallic acid equivalents.

The effects of DPA on soybean leaf TPC and genistin levels were analyzed. *Xcg* inoculation in the absence of DPA (0 μg/mL DPA) did not significantly alter soybean leaf TPC compared to the control leaves (Figure 10A). However, 50, 100, 200, and 500 μg/mL DPA increased TPC on days 1, 2, and 3 after pathogen inoculation in both preventive and curative assays. For example, in the preventive application, 500 μg/mL DPA increased TPC by 58.9%, 95.7%, and 134.0% on days 1, 2, and 3 after pathogen inoculation, respectively, compared to the control group. The increases in TPC were similar after application of 50, 100, 200, and 500 μg/mL DPA, suggesting that the effects are not dependent on DPA concentration. Although application of 50 μg/mL copper(II) chloride slightly increased TPC on days 1, 2, and 3 after pathogen inoculation, the effects caused by copper(II) chloride were not as large in magnitude as the effects caused by DPA.

Consistent with the increase in TPC, DPA application also increased genistin level at day 3 after pathogen inoculation (Figure 10B). The increase was detected in both preventive and curative applications. In preventive application, 50, 100, 200, and 500 μg/mL DPA increased genistin level by 22.9%, 21.3%, 24.4%, and 23.2%, respectively, compared to the control group. Similarly, in curative application, 50, 100, 200, and 500 μg/mL DPA increased genistin level by 45.5%, 44.7%, 42.1%, and 54.4%, respectively, compared to the control group. The application of 50 μg/mL copper(II) chloride did not significantly affect genistin level. Although the isoflavone Biochanin A has been reported to exhibit strong antibacterial activity against *Xcg* [21], Biochanin A was not detected in the soybean leaf extracts (under the detection limit).

### 3.9. High DPA Concentrations Show Toxicity in the Zebrafish Model

**Figure 11:**
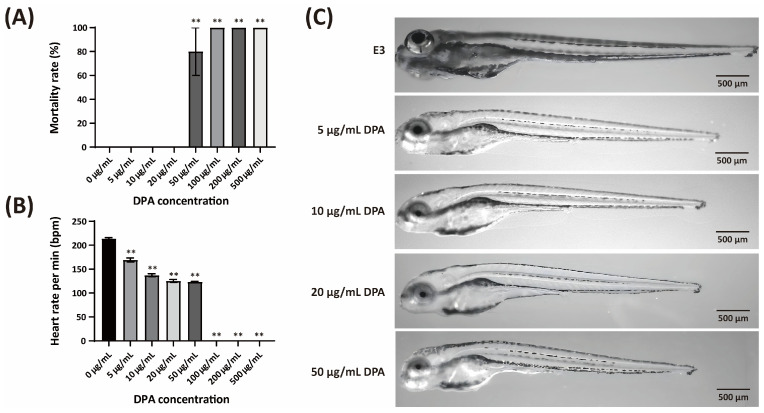
Developmental toxicity of DPA in zebrafish embryos. Zebrafish embryos were exposed to DPA at concentrations of 0, 5, 10, 20, 50, 100, 200, and 500 μg/mL from 4 h post-fertilization (hpf) to 4 days post-fertilization (dpf). (**A**) Mortality rate at 4 dpf. Data represent mean ± SD from three independent replicates (20 embryos in each batch, with 3 repetitions (n = 60)). (**B**) Heart rate of surviving embryos at 4 dpf, expressed as beats per minute (bpm). Data represent mean ± SD (n = 60 larvae per DPA concentration). (**C**) Representative images of zebrafish embryos at 4 dpf. Compared with the control group (0 μg/mL), embryos treated with 10 and 20 μg/mL DPA exhibited reduced body length. No embryos survived at concentrations ≥100 μg/mL. Significance levels: ** *p* ≤ 0.01. Scale bar = 500 μm.

The developmental toxicity of DPA was evaluated using zebrafish embryos exposed continuously from 4 h post-fertilization (hpf) to 4 days post-fertilization (dpf). As shown in Figure 11A, no mortality was observed at DPA concentrations of 0, 5, 10, and 20 μg/mL. However, exposure to 50 μg/mL DPA resulted in 80% mortality, and concentrations of 100, 200, and 500 μg/mL DPA caused complete mortality (100%) before 4 dpf, indicating a sharp lethal threshold between 20 and 50 μg/mL DPA.

Heart rate was measured in surviving embryos at 4 dpf (Figure 11B). In the control group (0 μg/mL DPA), the heart rate was 214 ± 2 bpm. Treatment with 5 and 10 μg/mL DPA significantly reduced the heart rate to 169 ± 4 bpm and 137 ± 3 bpm, respectively. At 20 μg/mL DPA, the heart rate further decreased to 125 ± 3 bpm. Although a few larvae survived at 50 μg/mL DPA, their heart rate (123 ± 1 bpm) remained similarly reduced; however, the limited number of survivors precluded robust statistical comparison.

In addition to mortality and bradycardia, DPA exposure caused concentration-dependent morphological abnormalities. Representative images taken under the same magnification (Figure 11C) showed that larvae treated with 10 and 20 μg/mL DPA exhibited a markedly reduced body length compared to the control group. Furthermore, larvae exposed to 20 μg/mL DPA frequently displayed a curved or wavy spinal axis (spinal curvature), indicating that DPA not only retards growth but also induces skeletal malformations. These phenotypic changes were not obvious in the control group or in larvae treated with 5 μg/mL DPA.

Taken together, these results demonstrate that DPA exhibits dose-dependent developmental toxicity in zebrafish, with significant lethal effects at concentrations ≥50 μg/mL.

## 4. Discussion

Although DPA has previously been reported to exhibit antibacterial activity against certain human pathogenic bacteria [39,40], this study provides the first comprehensive investigation of its antibacterial mechanism against a plant-pathogenic bacterium, *Xcg*, and demonstrates that it is a metabolite contributing to the antibacterial activity of *Bacillus* and *Paecilomyces* [67,68]. DPA showed slightly lower antibacterial activity compared to the commercial bactericides kanamycin and streptomycin sulphate, confirming its potential for practical application. A previous study engineered *B. subtilis* by replacing the *spoVF* operon promoter to enhance DPA production [31]. Our findings demonstrated that 500 μg/mL DPA does not show antibacterial activity against *B. subtilis*. Thus, optimizing DPA production in *B. subtilis* by modifying *B. subtilis* culture media or via *B. subtilis* genetic manipulation appears to be a suitable strategy to enhance the antibacterial activity of *B. subtilis* cell-free supernatant.

Lipopeptides and volatile organic compounds (VOCs) produced by *Bacillus* exhibit antibacterial properties. For example, *Bacillus* sp. LLB-04-produced iturin C3 showed antibacterial activity against *Listeria monocytogenes* MTCC 657, *Streptococcus mutans* MTCC 890, *S. aureus* MTCC 96, and *Salmonella enterica* serovar Typhimurium MTCC 98, with MIC values ranging from 0.1 to 1.6 mg/mL [69]. Additionally, the cyclic lipopeptide surfactin synthesized by *B. subtilis* natto NTU-18 inhibited biofilm formation by *Enterococcus faecalis* [70]. *Bacillus halotolerans* NYG5-emitted VOCs inhibited the growth of various plant bacterial pathogens, including *Agrobacterium tumefaciens*, *X. campestris*, *Clavibacter michiganensis*, and *Pseudomonas syringae* [71]. Although *Paecilomyces* strains have been mainly used for the management of plant pathogenic nematodes [72], a few reports have also shown that *Paecilomyces* strains can be used for the management of fungal diseases in plant roots and for the control of fungal blights and vascular diseases [67]. As far as we know, no report regarding the potential antibacterial activity of *Paecilomyces* strains can be found in the literature. Given that *Bacillus* and *Paecilomyces* species are commonly found in soil [73,74,75,76], it is possible that DPA also plays a key role in their interactions with other soil microorganisms.

The EC_50_ of pABA against *Xcg* was 4.02 mM (551.3 μg/mL), which is much higher than that observed with DPA (53.2 μg/mL) [23]. Both DPA and genistin showed an MIC value against *Xcg* of 500 μg/mL [22]. Instead, Biochanin A showed an MIC against *Xcg* as low as 100 μg/mL [21]. DPA, priced at approximately 0.79 dollars per gram, is considerably less expensive than genistin, which costs around 0.88 dollars per milligram, and Biochanin A, priced at approximately 71.23 dollars per gram. This cost differential suggests that DPA may be more suitable for managing bacterial pustule disease compared to the specified isoflavones. The strong activity of DPA against *Xcg* but not against other *Xanthomonas* pathovars suggests that *Xcg* must have some specific characteristics that make it more susceptible to DPA than other pathovars. It would be interesting to study in depth the antibacterial mechanism of DPA against *Xcg* to understand this singularity. It must be highlighted that *X. citri* has some membrane singularities, unique proteins, such as XAC1347 (OMP*_Xan_*) and TolB, which are not found in other *Xanthomonas* species [77]. This may explain the specific antibacterial activity of DPA against *Xcg* but not against the other pathovars used in this study. However, further research is necessary to confirm that these membrane proteins are involved in the antibacterial mechanism. Although the narrow-spectrum activity of DPA may limit its commercial potential, selective antibacterial agents offer the advantage of targeting the pathogen without harming beneficial microorganisms.

Swimming motility, extracellular protease activity, and biofilm formation are key virulence factors of *Xcg* [9,11]. The antibacterial metabolites pABA and Biochanin A have also been reported to reduce *Xcg* swimming motility, extracellular protease activity, and biofilm formation [21,22]. It has been reported that the biofilm protects *Xanthomonas* from being recognized by plant host cells and assists in the adhesion of *Xanthomonas* to the host surface [78].

In addition to the effects on biofilm formation, DPA also altered *Xcg* membrane integrity. Similarly, application of pABA also resulted in altered morphology and *Xcg* cell lysis [23]. This effect was attributed to pABA-induced alterations in the profiles of OMPs and LPS, which play a key role in the outer membrane integrity of Gram-negative bacteria [23]. Genes *342RT*, *1501RT*, and *2689RT* encode RND efflux system membrane fusion-like proteins (CmeA) [64]. DPA reduced the expression of the efflux system proteins CirA, CzcA, CzcB, and EmrE, which are also OMPs and play a key role in the outer membrane integrity by maintaining the osmotic balance [9,60,61]. KefB is a membrane component involved in K^+^ transport [63], while TolC is an outer membrane efflux protein that contributes to the secretion of proteins, polysaccharide biosynthesis, and antimicrobial resistance [62]. Similarly, pABA application also inhibited the expression of these proteins [23]. Within *Xanthomonas*, the *ftsZ* gene is indispensable for cell division [79]. Targeting the FtsZ protein of *X. oryzae* pv. *oryzae* with antibacterial compounds has been pursued as a viable strategy to combat this rice pathogen. In *Xanthomonas* cell division, ZapA stabilizes FtsZ [80], whereas MinD acts as an inhibitory regulator [81]. Among the genes associated with *Xcg* pathogenicity, *yapH* has been shown to be essential for full virulence [64]. Additionally, the transporter genes *yapH* and *1578RT*, along with the regulatory gene *rpfE*, contribute to the ability of *Xcg* to colonize soybean leaves. Although DPA promoted cell division gene expression and did not obviously affect pathogenesis-related genes, further research is necessary to understand its effects on stress-response genes (oxidative stress, metabolism, or quorum-sensing genes) for a more comprehensive mechanistic insight. DPA is a well-known chelator. Iron has been reported to play a key role in the stability of the *Xanthomonas* inner and outer membrane [82]. Furthermore, iron availability and uptake are necessary to promote diffusible signal factor (DSF) biosynthesis, which regulates biofilm formation and motility [82]. Therefore, the effects caused by DPA may be mediated through its chelating properties and the consequent impact on iron availability.

DPA showed one of the highest efficacies among the reported approaches for soybean bacterial pustule disease management. Preventive application of 500 μg/mL DPA reduced lesion numbers by 82.7%, which is comparable to the 89.0% reduction achieved with Biochanin A at 500 μg/mL [21] and the 95.5% reduction reported for a thymol nanoemulsion [83]. However, the practical applicability of these agents differs substantially. Biochanin A is prohibitively expensive for agricultural use (approximately 71.23 dollars per gram), while thymol is more affordable (approximately 10 dollars per kilogram) but requires nanoformulation to achieve high efficacies. In contrast, DPA is commercially available at approximately 0.79 dollars per gram, making it considerably more cost-effective than Biochanin A and comparable in price to thymol without requiring specialized formulation. When compared with other natural compounds, DPA demonstrates superior efficacy relative to its application concentration. For instance, pABA required 1371 μg/mL to achieve only 47.5% disease reduction [23], whereas DPA achieved 82.7% reduction at 500 μg/mL, indicating a more favorable dose–response profile. Genistein applied via soil irrigation at 50 μg/mL reduced lesions by 63.0% [22], but this approach requires continuous soil application rather than foliar spraying. Furthermore, the efficacy of DPA exceeds that of copper-based bactericides (31.9% reduction) and compares favorably with the commercial antibiotics oxytetracycline (74.1% reduction) and streptomycin (65.0% reduction) [84] while avoiding the human health and environmental concerns associated with antibiotic use in agriculture [85,86].

Beyond its potential for standalone application, our results confirmed that DPA contributes significantly to the antibacterial activity of the biocontrol agents *Bacillus* and *Paecilomyces*. A key point to consider is that *B. subtilis* 168 is unable to synthesize lipopeptides [31]. For this reason, DPA may play a more important role in the antibacterial activity of this strain than in other strains. Furthermore, the cell-free supernatant of *P. maximus* NJC01 reduced *Xcg* growth to a greater extent than that of *B. subtilis*, suggesting that *Paecilomyces* strains may have potential for the control of plant bacterial pathogens.

A recent report indicates that DPA induces disease resistance in kiwifruit against *B. cinerea* by promoting the accumulation of antifungal phenolics [33]. DPA was found to increase TPC and genistin content in *Xcg*-infected soybean leaves, corroborating the previous finding. Genistin has been shown to exhibit significant antibacterial activity against *Xcg* (MIC = 500 μg/mL) [22], suggesting that its accumulation in soybean leaves may enhance soybean leaf resistance to bacterial pustule disease. Genistin is one of the most abundant flavonoids in soybean [87]. Previously, irrigation of soybean plants with genistein was reported to induce the biosynthesis of genistin, inducing disease resistance to *Xcg* [22]. Isoflavones were reported to play a key role in soybean resistance to the bacterial blight pathogen *Pseudomonas savastanoi* pv. *glycinea* [88]. The development of transgenic plants exhibiting increased isoflavone production has been identified as an effective method to enhance the intrinsic resistance of legume plants to pathogen infections [89,90]. These results demonstrate that DPA application is also an effective strategy for enhancing soybean isoflavone content.

The safety profile of any agricultural chemical is critical for its practical application. In this study, we evaluated the developmental toxicity of DPA using the zebrafish model, a widely accepted vertebrate system for chemical safety assessment. Our results showed that DPA concentrations up to 20 μg/mL did not cause mortality in zebrafish embryos. Notably, the effective concentration of DPA for controlling *Xcg* in soybean leaves was 100 μg/mL, which is 5-fold higher than the highest non-lethal concentration (20 μg/mL) observed in zebrafish. Considering the typical dilution ratios when compounds are applied in the field, the concentration of DPA reaching aquatic ecosystems is expected to be significantly lower than 20 μg/mL. This suggests that DPA may have a favorable safety profile for agricultural use. When comparing the mortality rates induced by DPA with those of other commonly used pesticides in the zebrafish model, DPA exhibited very weak toxicity. For example, treatment with 0.001 μg/mL carbendazim caused mortality rates higher than 70%, while 10 μg/mL ametryn resulted in 100% mortality [91]. Similarly, linuron at 2 μg/mL caused 100% mortality [92]. These comparisons suggest that DPA may have the potential to meet the safety requirements for commercialization.

## 5. Conclusions

Collectively, the antibacterial properties of DPA, a metabolite present in the biocontrol agents *Bacillus* and *Paecilomyces*, were examined for the first time against pathogenic *Xanthomonas* pathovars. The results demonstrate that DPA shows strong antibacterial activity against *Xcg*, with an EC_50_ of 53.2 µg/mL, but no significant antibacterial activity against other *Xanthomonas* pathovars. DPA reduced *Xcg* swimming motility, extracellular protease activity, biofilm formation, and biofilm reducing sugar content. DPA altered *Xcg* membrane integrity, inducing cell lysis, and reduced the expression of various OMPs, including CirA, CzcA, CzcB, EmrE, and TolC. DPA showed both preventive and curative efficacies. The application of 500 μg/mL DPA reduced the number of lesions caused by *Xcg* in preventive and curative applications by 82.7% and 83.8%, respectively, demonstrating one of the highest efficacies for *Xcg* management reported in the literature. DPA enhanced TPC and genistin levels in *Xcg*-infected soybean leaves. DPA exhibited only weak toxicity in the zebrafish model, suggesting that it is safe for agricultural use. This study reveals the potential of DPA for managing bacterial pustule disease in soybean and provides new insights into an underexplored antibacterial metabolite from the biocontrol agents *Bacillus* and *Paecilomyces*. The findings suggest that DPA acts through a dual mechanism involving direct antibacterial activity against *Xcg*, including inhibition of swimming motility, biofilm formation, and membrane integrity, and induction of plant defense responses via accumulation of the antibacterial isoflavone genistin. DPA exhibits efficacy comparable to commercial antibiotics while avoiding human health and environmental concerns, and its low cost and commercial availability make it a promising candidate for further development. Additionally, DPA represents a potential target for enhancing the efficacy of *Bacillus*- and *Paecilomyces*-based biocontrol products through strain engineering. Further research is necessary to understand why DPA shows antibacterial activity against *Xcg* but not against other *Xanthomonas* pathovars.

## Figures and Tables

**Table 1:** Inhibitory activity of DPA against various *Xanthomonas* pathovars and *Bacillus subtilis* ^1^.

Bacterial Strains	EC_50_ (μg/mL) ^2,3^	MIC (μg/mL) ^2,4^
DPA		
*Xanthomonas campestris* pv. *campestris* FE58	476.5 ± 8.6	>500
*Xanthomonas citri* pv. *glycines* ICMP5732	53.2 ± 3.5	500
*Xanthomonas oryzae* pv. *oryzae* PXO99	>500	>500
*Xanthomonas oryzae* pv. *oryzicola* RS105	448.6 ± 9.3	>500
*Bacillus subtilis* 168	>500	>500
Copper(II) chloride		
*Xanthomonas citri* pv. *glycines* ICMP5732	123.3 ± 5.6	>500
Kanamycin		
*Xanthomonas citri* pv. *glycines* ICMP5732	4.2 ± 0.7	20
Streptomycin sulphate		
*Xanthomonas citri* pv. *glycines* ICMP5732	11.2 ± 1.6	20
Tetracycline		
*Xanthomonas citri* pv. *glycines* ICMP5732	0.9 ± 0.2	10

^1^ *Xanthomonas* growth was monitored according to the OD_600_ value. ^2^ The bactericides were used at 0, 5, 10, 20, 50, 100, 200, and 500 μg/mL in the assay. ^3^ Refers to the concentration of the bactericide that reduced bacterial growth by 50%. ^4^ Refers to the concentration of bactericide that completely inhibited bacterial growth. Abbreviations: DPA, dipicolinic acid (2,6-pyridinedicarboxylic acid); EC_50_, half-maximal effective concentration; MIC, minimum inhibitory concentration.

**Table 2:** Efficacy of DPA for the control of bacterial pustule disease symptoms.

Application	Treatment	Number of Lesions per Leaf ^1,2^				Inhibitory Activity (%) ^3^
		After 1 Day	After 2 Days	After 3 Days	After 4 Days	
Preventive application	Control ^4^	3.8 ± 1.5 ^a^	32.1 ± 7.2 ^a^	62.7 ± 9.9 ^a^	124.7 ± 6.8 ^a^	-
	50 μg/mL DPA	3.2 ± 1.0 ^a^	23.9 ± 5.6 ^b^	58.8 ± 10.3 ^a^	91.1 ± 11.5 ^b^	26.9
	100 μg/mL DPA	2.7 ± 1.6 ^a^	22.0 ± 5.5 ^b^	47.9 ± 2.5 ^b^	60.9 ± 3.6 ^c^	51.2
	200 μg/mL DPA	2.2 ± 2.1 ^a^	16.3 ± 1.7 ^b^	26.2 ± 5.7 ^c^	44.8 ± 5.6 ^d^	64.1
	500 μg/mL DPA	1.3 ± 1.3 ^a^	9.5 ± 2.1 ^c^	16.3 ± 1.8 ^d^	21.6 ± 1.4 ^e^	82.7
	Copper(II) chloride ^5^	1.0 ± 0.9 ^a^	9.5 ± 1.0 ^c^	14.1 ± 2.0 ^d^	21.6 ± 2.2 ^e^	82.7
Curative application	Control ^4^	2.6 ± 0.6 ^a^	11.2 ± 1.5 ^a^	42.0 ± 6.0 ^a^	152.1 ± 8.9 ^a^	-
	50 μg/mL DPA	2.1 ± 1.0 ^a^	7.0 ± 1.7 ^b^	35.5 ± 4.2 ^ab^	109.3 ± 9.1 ^b^	28.1
	100 μg/mL DPA	1.7 ± 1.6 ^ab^	6.9 ± 2.1 ^b^	31.2 ± 1.5 ^b^	103.1 ± 4.4 ^b^	32.2
	200 μg/mL DPA	1.2 ± 0.9 ^ab^	5.1 ± 1.0 ^b^	25.9 ± 2.0 ^c^	51.0 ± 16.6 ^c^	66.5
	500 μg/mL DPA	1.0 ± 1.0 ^ab^	1.8 ± 1.5 ^c^	15.3 ± 2.1 ^d^	24.6 ± 3.2 ^d^	83.8
	Copper(II) chloride ^5^	0.4 ± 0.3 ^b^	1.7 ± 1.5 ^c^	15.2 ± 6.8 ^d^	18.0 ± 5.6 ^d^	88.2

^1^ Each treatment mean represents the average of 150 leaves ± standard deviation (SD). ^2^ Differences between means in the same column were considered significant when *p* ≤ 0.05. Different letters indicate that the means were statistically different. The curative and preventive assays were considered two different statistical groups. ^3^ Inhibitory activity was calculated based on data collected 4 days post-inoculation. ^4^ The control experiment was performed in the absence of antibacterial agents. ^5^ Copper(II) chloride, CuCl_2_, was employed at 50 μg/mL concentration. Abbreviations: DPA, dipicolinic acid (2,6-pyridinedicarboxylic acid).

## Data Availability

The original contributions presented in this study are included in the article/Supplementary Materials. Further inquiries can be directed to the corresponding authors.

## Supplementary Materials

The following supporting information can be downloaded at: https://www.mdpi.com/article/10.3390/biom16040605/s1, Table S1: Reagents used in the study; Table S2: Primers used in the qRT-PCR analysis; Figure S1: Microscopic observations of DPA-treated *Xanthomonas citri* pv. *glycines* (*Xcg*) cells after staining with crystal violet and safranine. Control cells were untreated, while treated cells were exposed to 100 μg/mL DPA; Figure S2: Survival of *Xanthomonas citri* pv. *glycines* (*Xcg*) cells following DPA treatment. Control cells were untreated, whereas treated cells were exposed to 50, 100, or 500 μg/mL DPA. DPA induced membrane abnormalities in *Xcg* cells. However, when cell lysis did not occur, the cells resumed growth. Scale bar = 10 μm.

## Abbreviations

The following abbreviations are used in this manuscript:

bpm	Beats per minute
CFU	Colony-forming units
ddH_2_O	Double-distilled water
dpf	Days post-fertilization
DSF	Diffusible signal factor
DNS	3,5-Dinitrosalicylic acid
DPA	Dipicolinic acid
EC_50_	Half-maximal effective concentration
GAE	Gallic acid equivalents
H	Hours
hpf	Hours post-fertilization
LB	Luria–Bertani
LPS	Lipopolysaccharide
MIC	Minimum inhibitory concentration
Min	Minutes
OMP	Outer membrane protein
pABA	*p*-Aminobenzoic acid
PDA	Potato-dextrose agar
PDB	Potato-dextrose broth
qRT-PCR	Quantitative real-time PCR
RND	Resistance-nodulation-cell-division
rpm	Revolutions per minute
SEM	Scanning electron microscope
TEM	Transmission electron microscope
TPC	Total phenolic content
VOCs	Volatile organic compounds
*Xcg*	*Xanthomonas citri* pv. *glycines*
