# Supplementary material for: Antibacterial Mechanism of Dipicolinic Acid Against Xanthomonas citri pv. glycines and Its Efficacy for the Management of Soybean Bacterial Pustule Disease
Source: Biomolecules. 2026 Apr 19;16(4):605. doi: 10.3390/biom16040605 (PMC13113452; doi:10.3390/biom16040605)
Supplement: Supplementary file 1 [file biomolecules-16-00605-s001.zip › biomolecules-4223959-supplementary.pdf]

Supplementary Material

Antibacterial Mechanism of Dipicolinic Acid against *Xanthomonas citri* pv. *glycines* and Its Efficacy for the Management of Soybean Bacterial Pustule Disease

Lei Chen [1,#], Jia-Xuan Shen [1,#], Ming-Yi Zhang [1,#], Xin-Chi Shi [1], Lei Xu [2], Si-Yuan Liu [3], Daniela D. Herrera-Balandrano [4], Pere Clapés [5], Jie Gong [1], Dong Liu [1], Su-Yan Wang [1,*] and Pedro Laborda [1,*]

[1] School of Life Sciences, Nantong University, Nantong 226019, China

[2] Core Facilities, Faculty of Health Science of Nantong University, Nantong 226019, China

[3] Nantong University Analysis & Testing Center, Nantong University, Nantong 226019, China

[4] Department of Food and Human Nutritional Sciences, University of Manitoba, Winnipeg R3T 2N2, Canada

[5] Department of Biological Chemistry, Institute for Advanced Chemistry of Catalonia (IQAC-CSIC), Barcelona 08034, Spain

[*] Correspondence: pedro@ntu.edu.cn (P.L.); wangsuyan@ntu.edu.cn (S.-Y.W.).

Table S1. Reagents used in the study.

Reagent	Product number	Supplier; Country
DPA	P816098	Macklin; China
Tryptone	LP0042B	Oxoid; China
Yeast extract	LP0021	Oxoid; China
Sodium chloride	-	Xihua, China
Agar powder	-	Solarbio; China
Methanol (≥99.5% purity)	G75851L	Shanghai Titan Scientific; China
Glacial acetic acid	-	Richjoint; China
Hydrochloric acid (36.0%-38.0% *v/v*)	-	Richjoint; China
Ethanol (≥99.7% purity)	G73537B	Shanghai Titan Scientific; China
Crystal violet	C805209-100g	Macklin; China
Potassium iodide	GBT1272-2007	Shanghai Shenbo Chemical; China
Iodine (99.8% purity)	1812016-25g	Macklin; China
Safranine T	S817532-10g	Macklin; China
Ammonium acetate	A800996-500g	Macklin; China
DNS	D807260-25g	Macklin; China
Glucose	G6172	Macklin; China
p-Formaldehyde (37.0%-40.0% *v/v*)	-	Xilong Scientific; China
Glutaraldehyde	AFIHC049	AiFang Biological; China
Uranyl acetate	NC1630601	Fisher Scientific; USA
Lead citrate (3% *w/v*)	22410	Electron Microscopy Sciences; USA
Folin-Ciocalteu reagent	P824172	Macklin; China
Gallic acid (99% purity)	G823163	Macklin; China
Genistin (10 mM in DMSO)	T2882	Targetmol; China
Calcofluor-white	18909	Sigma-Aldrich; USA
Copper(II) chloride dihydrate ($CuCl_2 \times 2H_2O$)	C805298-500g	Macklin; China

Abbreviations: DMSO, dimethyl sulfoxide; DNS, 3,5-dinitrosalicylic acid; DPA, dipicolinic acid (2,6-pyridinedicarboxylic acid).

Table S2. Primers used in the qRT-PCR analysis [1].

Gene name	Description	Forward primer	Reverse primer
**Reference gene**			
16S rRNA	Reference gene	TAACGACGCCACTTATGCAA	GCCAATCCCAGAAACCCTAT
**Membrane integrity-related genes**			
342RT	RND efflux system, membrane fusion protein (CmeA)	CAAGCGCCTGTTCACCGAGG	GAGATTGATGCGCGCGGTCT
1501RT	RND efflux system, membrane fusion protein (CmeA)	AACTGTCCGAGCAGCAGGC	GACGAACACCTTGTCCAGCGA
2689RT	RND efflux system, membrane fusion protein (CmeA)	GCCAAGGTATCGCTGCTGCT	GCACGCAACTGCAGCTTGTG
cirA	Outer membrane receptor protein, mostly Fe transport	TTGACGGTGAAGCCGGTGAT	CTATCTATGCCGAACTCCAAGCCT
czcA	Cation efflux system protein	TTGGGATTTACCCTGGACGACG	GTCAAAGACCACGCCCACGG
czcB	Cation efflux system protein	GGGCGTCACCGAGGTCAACA	CTAAATGGGACCTGCTGCAACGG
emrE	Membrane transporter of cations and cationic drug	GCAGGCGCTGAAGACCATTC	TAAGCCGCTATCGCGGAGCA
kefB	Kef-type K^+ transport system, membrane component	ATTGGAGCGTTGCGTTCTTGC	CGTTTGCGGTCGGCAGGCAT
tolC	Outer membrane protein	CAACCATCGACAAGATCGAAATCA	AGACATACTCCGGCACCCGT
**Pathogenicity-related genes**			
1578RT	Phosphate transport, ATP binding protein (pstB)	AGCTTGTCCTTGACCTCGTCC	GTATCCGAAGATGGAAGCGCG
rpfE	Regulatory protein	TCAGACTGAAATGGCGCTGCAA	GGCATAGCGGGTCCCAAGGC
yapH	Autotransporter adhesin	GACGGTGCAGATCAACAATG	CCCGAGTTGACGAACGTATT
**Cell division-related genes**			
4495RT	Cell division topological specificity factor	CCTAATGGACGGCGACTACG	TCTCAAGAGCAAGAAGAACA
ftsA	Cell division protein FtsA	CCTCGCAGAACTCTACCTGC	GGGTGGTCCAGACAGCAAGA
ftsL	Cell division protein FtsL	CACCCGCCTTTCGTTGGCCC	TGCATCGCAAGCTGTTCGTG
ftsZ	Cell division protein FtsZ	CTTGGGCGACGACCTGCTAC	ACCGTGATGTCGGAAATGGG
minD	Septum site-determining protein MinD	AGTTGCGAAGGTTGTTCCCG	GAAGGCGGCGAGATGCTCAG
zapA	Cell division protein ZapA	GACCTGTGGAACTTAGCGGC	GGATCGTGTCGCGGTGCTGG
zipA	Cell division protein ZipA	CTAGCACAGCAACAAGCACC	CACAGAAGGCGTCGAGCAGA

Abbreviations: RND, resistance-nodulation-cell-division.

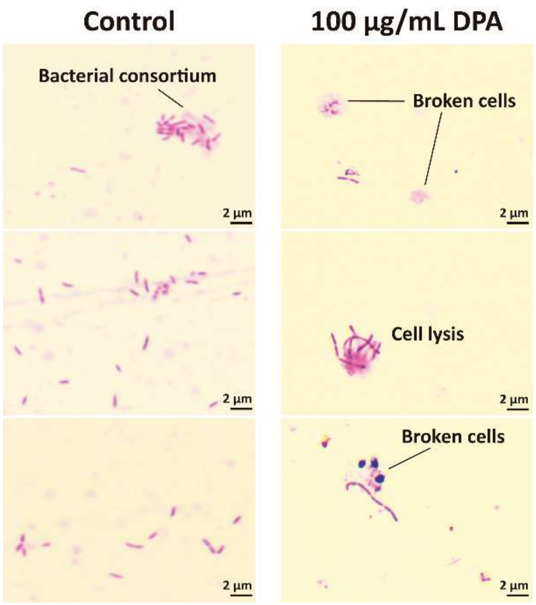

Figure S1. Microscopic observations of DPA-treated *Xanthomonas citri* pv. *glycines* (*Xcg*) cells after staining with crystal violet and safranine. Control cells were untreated, while treated cells were exposed to 100 µg/mL DPA.

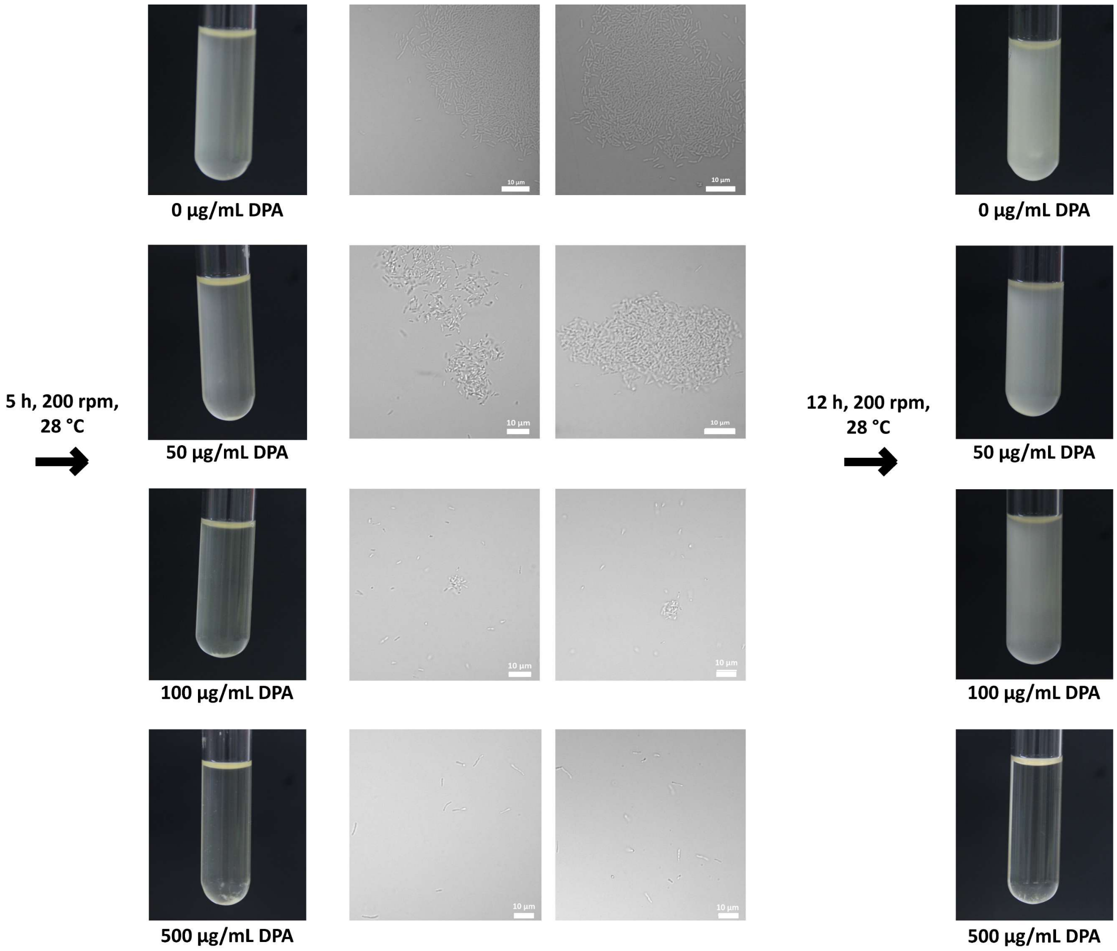

Figure S2. Survival of *Xanthomonas citri* pv. *glycines* (*Xcg*) cells following DPA treatment. Control cells were untreated, whereas treated cells were exposed to 50, 100, or 500 µg/mL DPA. DPA induced membrane abnormalities in *Xcg* cells. However, when cell lysis did not occur, the cells resumed growth. Scale bar = 10 µm.

References

1. Jiang, Y.H.; Liu, T.; Shi, X.C.; Herrera-Balandrano, D.D.; Xu, M.T.; Wang, S.Y.; Laborda, P. *p*-Aminobenzoic acid inhibits the growth of soybean pathogen *Xanthomonas axonopodis* pv. *glycines* by altering outer membrane integrity. *Pest Manag. Sci.* **2023**, *79*, 4083–4093.
